# Ovarian mature cystic teratoma with malignant transformation: two case reports

**DOI:** 10.1186/s13256-020-02594-4

**Published:** 2021-01-27

**Authors:** Afsaneh Tehranian, Akram Ghahghaei-Nezamabadi, Akram Seifollahi, Sara Kasraei, Hamideh Dehghani-Nejad, Arezoo Maleki-Hajiagha

**Affiliations:** 1 Department of Obstetrics and Gynecology, Arash Women’s Hospital, Tehran University of Medical Sciences, Rashid Ave, Resalat Highway, Tehranpars, P.O Box: 1653915981, Tehran, Iran; 2 Research Development Center, Arash Women’s Hospital, Tehran University of Medical Sciences, Tehran, Iran; 3 Pathology Department, Arash Women’s Hospital, Tehran University of Medical Sciences, Tehran, Iran

**Keywords:** Mature cystic teratomas, Dermoid cyst, Malignant transformation, Squamous cell carcinoma

## Abstract

**Background:**

Mature Cystic Teratoma (MCT) is a benign tumor that can lead to malignant transformation (MT) in 1–3% of cases. Management of MT is a big challenge for gynecologic oncologists due to the lack of specific diagnostic and treatment protocols.

**Case presentation:**

We reported two Iranian cases of MT of MCT with two different stages and prognosis. Our both cases presented the same symptoms, including chronic abdominal pain and distention, loss of appetite, and weight loss. In case number 1, despite the large size of the tumor, the disease was at stage Ia and had a good prognosis; while, case number 2 was at stage IIIc of the disease with a poor prognosis.

**Conclusion:**

The stage of the disease is the most important prognostic factor, and early diagnosis and treatment are very critical for better survival.

## Introduction

Mature cystic teratoma (MCT), a benign tumor commonly called the dermoid cyst, is the most common type of ovarian germ cell tumors. Over 80% of cases present in reproductive periods, especially in women under the age of 40. Malignant transformation (MT) is a rare and the worst complication of MCT that occurs in 1–3% of cases, especially postmenopausal women. Nearly 80% of the histological type of malignant transformations is squamous cell carcinoma (SCC), followed by adenocarcinoma, carcinoid tumor, melanoma, and sarcoma [1]. The preoperative-diagnosis is difficult because of non-specific findings; therefore, most patients are diagnosed in advanced stages and have poor outcomes. On the other hand, the diagnosis of MT of MCT at the early-stages is essential for longer survival. In the present study, we have described two cases of dermoid cysts with malignant transformation.

## Case presentation

### Case 1

**Fig. 1:**
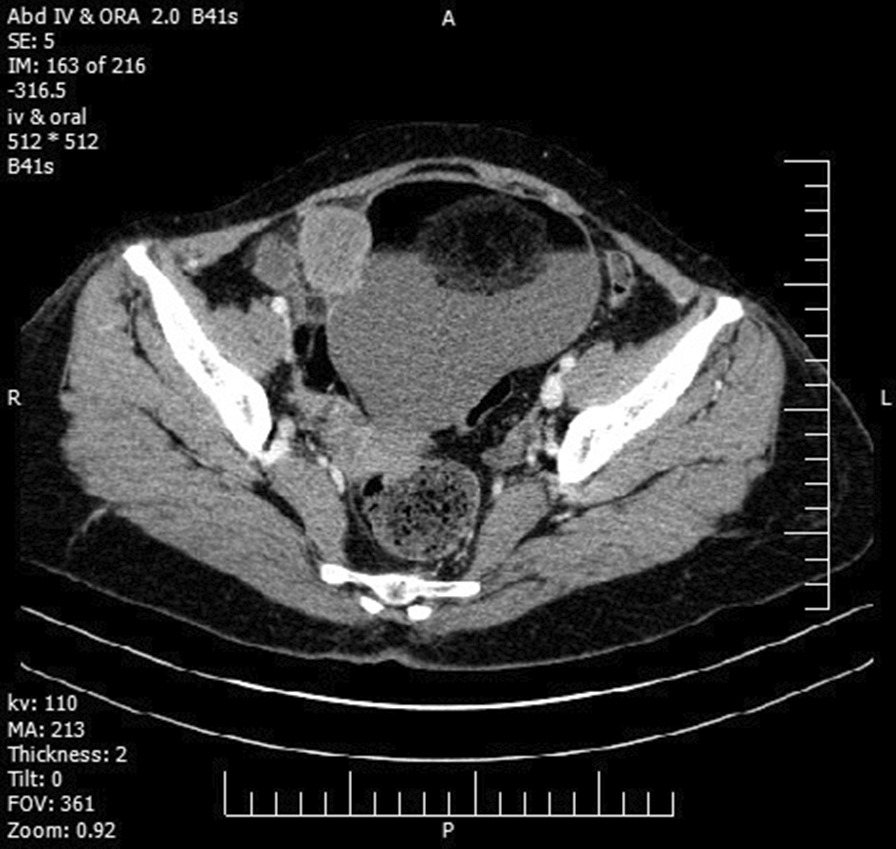
A contrast enhanced computerized tomography scan showing a complex large intra-abdominal solid-cystic lesion with intra-cystic enhanced fat component.

A 51-year-old, gravida 2, para 2, Iranian woman, suspected to have an ovarian tumor, was referred to our hospital. She complained of pelvic pain, anorexia, and weight loss of 10 kg over the past 3–4 months. The patient’s past medical, familial, social, environmental, and medication history was insignificant. The patient reported no history of smoking (cigarettes, tobacco, or shisha), drug abuse, or drinking alcohol. In all clinical examinations, except abdominopelvic examination, there were no significant findings, and the vital signs (including blood pressure, pulse rate, and body temperature) were normal. In abdominopelvic examination, a mass was palpated above the pelvis, from the midline to the lateral side, causing a mild compression effect against the rectum. All routine blood and urine laboratory tests were normal. The serum levels of cancer antigen 125 (CA125) and carcinoembryonic antigen (CEA) were above the normal ranges (129.4 u/ml and 34.7 ng/ml, respectively), while the amount of the other tumor markers, including human epididymis protein 4 (HE4), human chorionic gonadotropin (HCG), and alpha fetoprotein (AFP), were in the normal range (75.4 picomoles/l, 1.9 mIU/ml, and 1.2 ng/ml respectively). Abdominopelvic computed tomography (CT) scan showed a complex large solid-cystic (125 × 118 mm) mass with fat components, originated from the right adnexa, suggesting an ovarian dermoid cyst. This mass contained an oval-shaped, enhanced, solid component, sized 40 × 35 mm at its right lateral wall. Also, the CT scan detected an intrauterine device (IUD) in its normal location (Fig. 1).

After obtaining written informed consent, the patient underwent an exploratory laparotomy for staging and tumor debulking. Our chief surgeon opened the abdomen by a midline incision and performed peritoneal washing. The result of the cytological assessment of peritoneal washing was negative for malignancy, so the mass was completely excised and sent for frozen section analysis. Frozen section analysis reported a benign cystic teratoma. Then, the chief surgeon performed a total abdominal hysterectomy with bilateral salpingo-oophorectomy, omental biopsy, and pelvic lymph nodes sampling and sent the specimen for the histopathological examination.

**Fig. 2:**
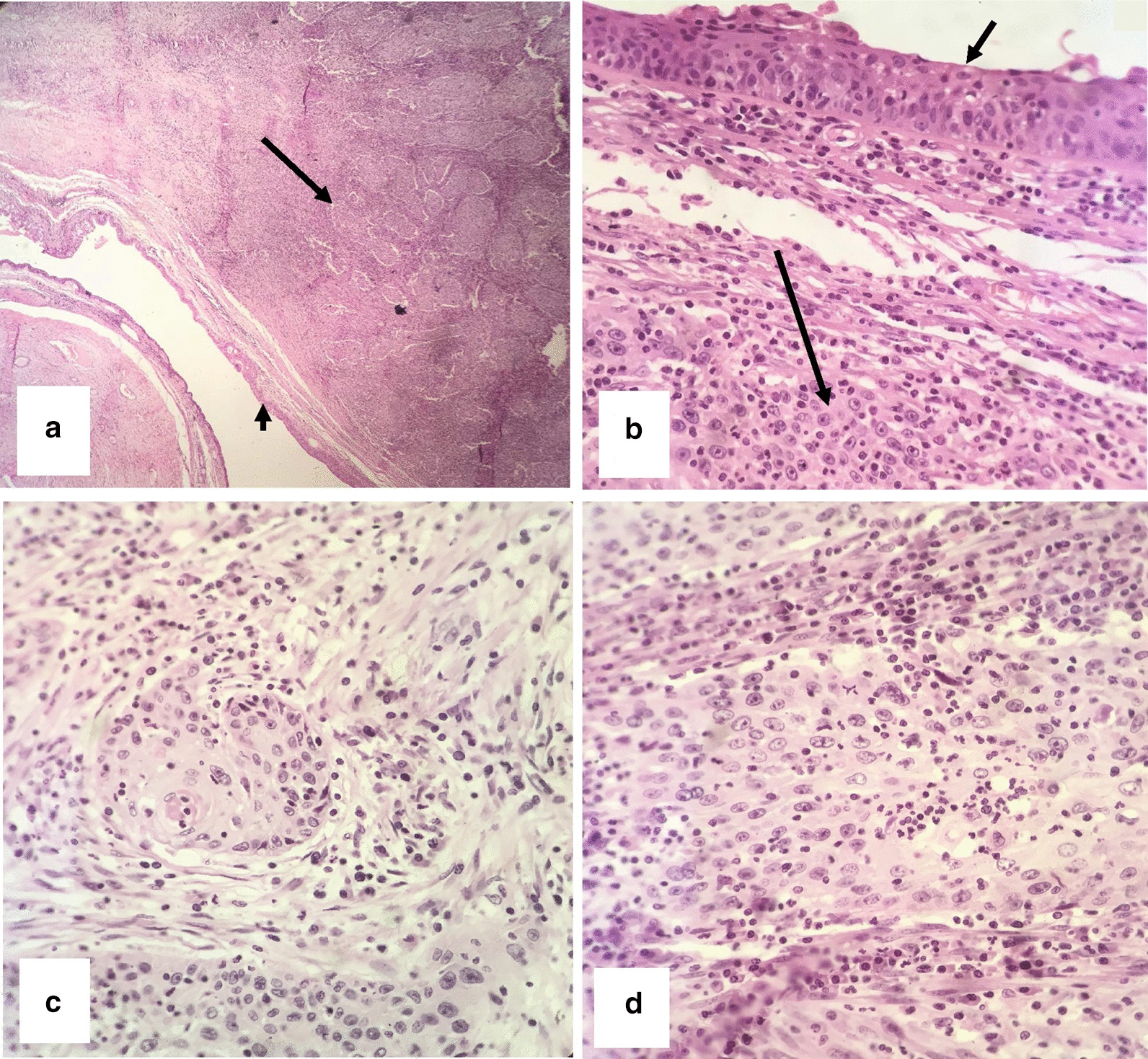
Microscopic findings of case number 1. **a** Haemotoxylin and eosin stained section shows a stratified squamous epithelium lining of mature cystic teratoma (short arrows) and nests of squamous cell carcinoma beneath the epithelium (long arrows) (×100). **b** Squamous epithelium (short arrows) and islands of squamous cell carcinoma (long arrows) (×400). **c**, **d** nests of moderately differentiated SCC (×400).

On gross examination, the surface of the right ovary was smooth and contained a mass with a size of 130 × 110 × 50 mm. On the cut section, a unilocular cyst filled with hair shaft and greasy brown material was observed. The cyst wall contained a solid tan mural nodule measuring 80 × 50 × 40 mm. Microscopic examination revealed stratified squamous epithelial lining of the cyst. Sections from the solid nodule showed moderately differentiated squamous cell carcinoma arising in MCT (Fig. 2). The rest biopsies, including ovarian serosa, right tube, uterus, left adnexa, omentum, and lymph nodes, were free of tumor. We classified the disease as stage 1a grade 2 according to the International Federation of Gynecology and Obstetrics (FIGO’s) classification. The patient underwent four courses of adjuvant chemotherapy (Paclitaxel 175 mg/m^2^ IV over 3 hours plus Carboplatin area under the curve (AUC) 5 intravenously, every 21 days).

We followed the patient for 6 months after the completion of chemotherapy with thoracic, abdominopelvic, and spinal CT scans. Due to the detection of multiple diverticula of the sigmoid colon, the patient immediately underwent a colonoscopy that was fortunately negative for malignancy. Also, we performed a bone scan for the patient that was in favor of vertebral lytic lesions. So, we considered a positron emission tomography/CT scan (PET/CT) for her that it also found no evidence in favor of metastasis. The results of the thoracic and pelvic CT scans were reported normal, and the levels of the CA125 (= 8.3 u/ml) and other tumor markers were in their normal ranges. So, we closely followed the patient during the last 20 months, and fortunately, no recurrence has been occurred until now.

### Case 2

**Fig. 3:**
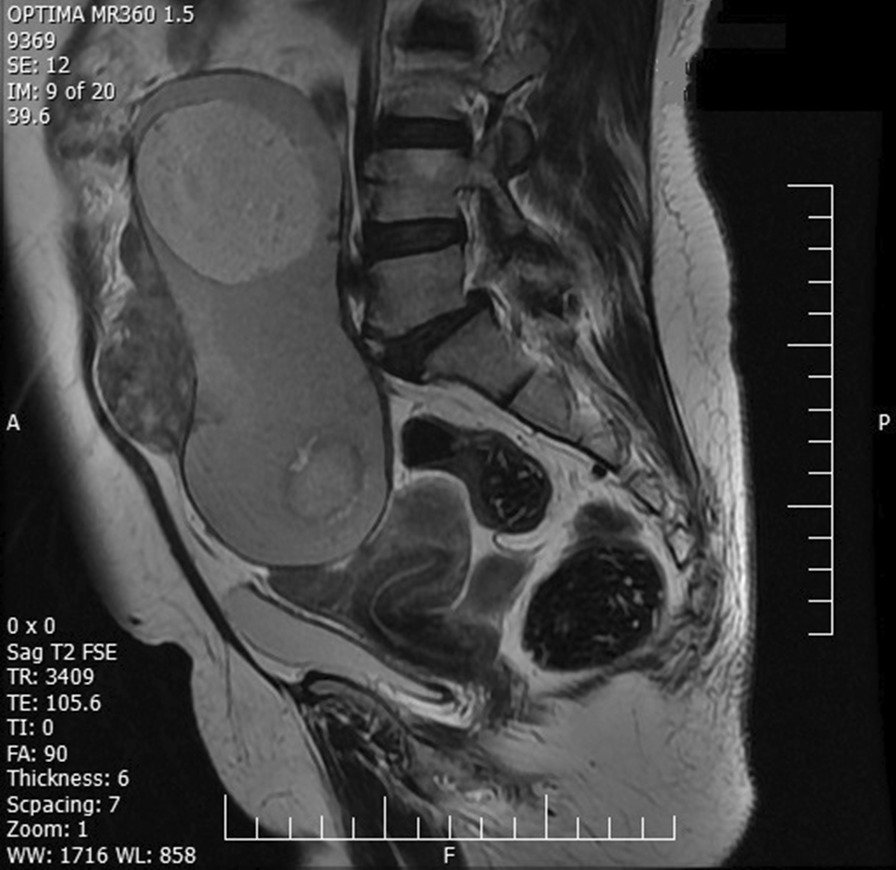
Sagittal T2-weighted fast spin echo magnetic resonance imaging image showing an intra-abdominal large solid-cystic mass, with fat foci and omental thickening in the vicinity of the tumor

**Fig. 4:**
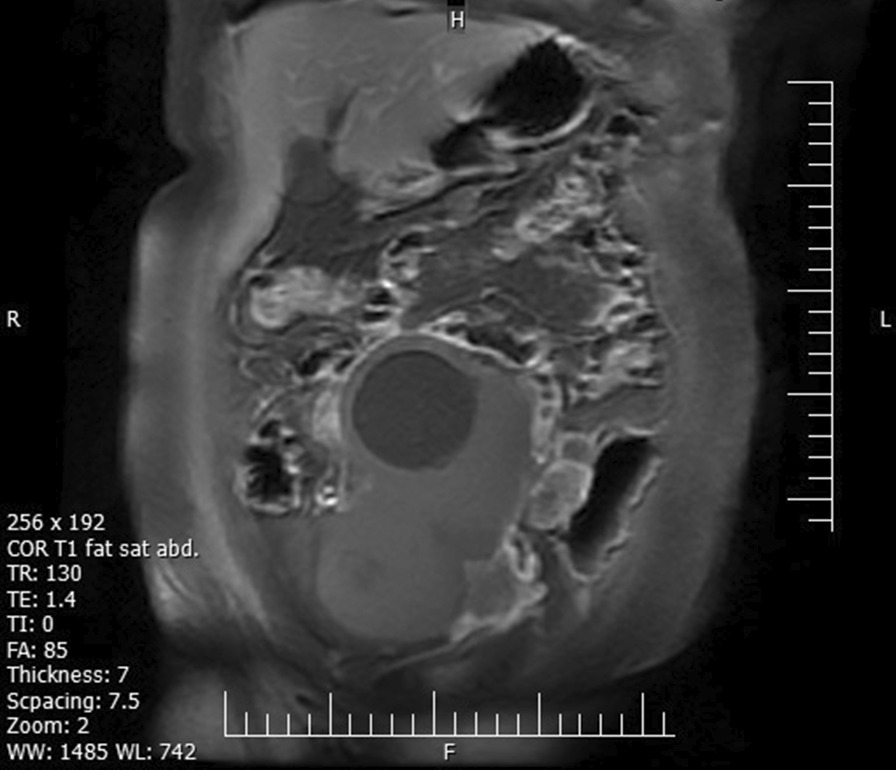
Coronal T1-weighted magnetic resonance imaging image with fat saturation showing an intra-abdominal large solid-cystic mass, with fat foci and omental thickening in the vicinity of the tumor

A 53-year-old, gravid 4, para4, postmenopausal Iranian woman with a suspicious ovarian tumor referred to our hospital’s oncologic clinic. She complained of chronic abdominal pain and distention, loss of appetite, and weight loss of 7–8 kg during the last 8 months. She has a history of chronic hypertension. The patient’s past familial, social, environmental, and medication history was insignificant. The patient reported no history of smoking (cigarettes, tobacco, or shisha), drug abuse, or drinking alcohol. In all clinical examinations, except abdominopelvic examination, there were no significant findings, and the vital signs (including blood pressure, pulse rate, and body temperature) were normal. On abdominopelvic examination, a mass was palpated that was extended from the pelvis to above the umbilicus. External genitalia and cervix had a normal appearance, and the Pap-smear test was negative for malignancy. The routine blood laboratory tests were within normal limits. Among the tumor markers, only the CA125 and ROMA were slightly elevated (40 u/ml and 38/47, respectively), and the rest were in the normal range. Ultrasound and magnetic resonance imaging scans showed a large solid-cystic intra-abdominal mass (sized 198 × 96 mm), with fat foci, originating from the right ovary, omental thickening in the vicinity of the tumor, and mild free fluid in the pelvis (Figs. 3, 4). The patient underwent laparotomy and surgical staging after obtaining written informed consent. Our chief surgeon made a vertical midline abdominal incision, and upon entering the abdomen, drowned 50 ml of yellow ascites fluid. The cytological evaluation of ascites fluid was negative for malignancy. After that, a large right-sided ovarian tumor with dense adhesion to the rectosigmoid omentum, abdominal wall, and bladder and was removed and sent for frozen section analysis. Then, we performed a total abdominal hysterectomy, infracolic omentectomy, and also pelvic and para-aortic lymph nodes biopsies.

**Fig. 5:**
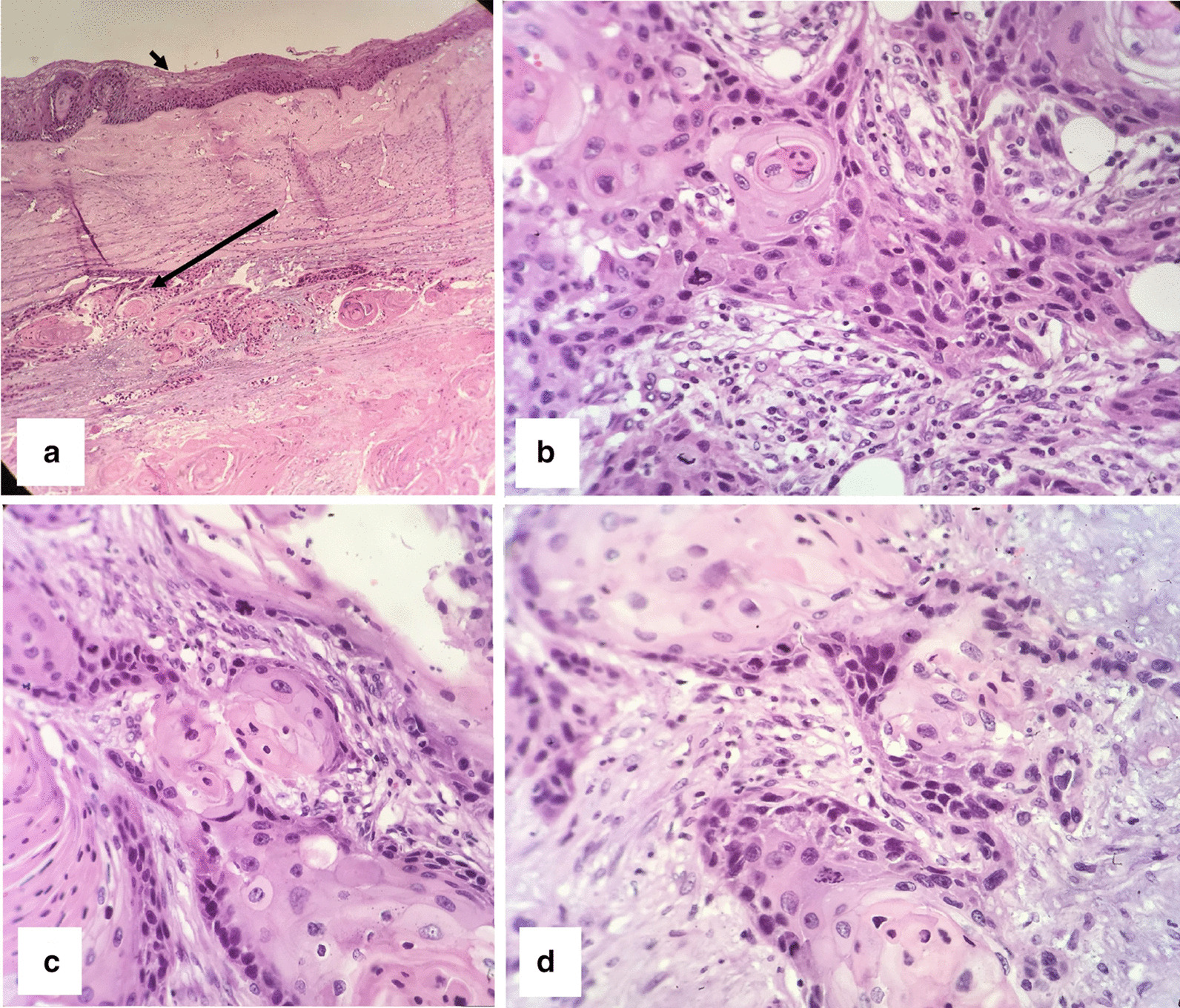
Microscopic findings of case number 2. **a** Haemotoxylin and eosin stained section shows a stratified squamous epithelium lining of MCT (short arrows) and nests of squamous cell carcinoma beneath the epithelium (long arrows) (×100). **b** Islands of squamous cell carcinoma with hyperchromatic pleomorphic nuclei and keratin pearl formation (×400). **c**, **d** Nests of SCC (×400).

Frozen section analysis reported teratoma with secondary malignant transformation, so we performed a complete surgical staging with cytoreductive surgery. In Macroscopic examination, the right ovary was a cyst measuring 140 × 90 × 90 mm containing hair and sebaceous material. Mural thickness was up to 30 mm. uterine serosa was irregular with visible tumoral masses. The histological examination revealed a well-differentiated SCC arising in mature cystic teratoma involving ovarian serosa, omentum, uterine serosa, myometrium pelvic serosa as retrovesical and rectosigmoid mass (Fig. 5). The pelvic and para-aortic lymph nodes were free of tumor. We staged the disease as IIIC according to the FIGO’s standard guidelines.

Four weeks after the operation, the patient underwent adjuvant chemotherapy with paclitaxel 175 mg/m^2^ IV over 3 hours and carboplatin AUC 5 IV every 3 weeks. Unfortunately, after completion of the third course of chemotherapy, she referred to our clinic with vaginal spotting. In the rectovaginal examination, a mass was palpated in the pelvis and upper part of the vaginal cuff, so she underwent an abdominopelvic MRI and a chest CT scan. A few days later, she presented signs of partial bowel obstruction. The chest CT scan was negative for malignancy, but the MRI showed a heterogeneous solid-cystic mass measuring 55 × 46 mm in the pelvic cavity, so we immediately performed an exploratory laparotomy. When we opened the abdominal wall, we found widespread peritoneal seeding of the tumor in the whole abdominal cavity and intestinal mesentery. We removed an ileal mass measured 50 × 60 mm and 3 weeks later second-line chemotherapy was started (gemcitabine 800 mg/m^2^ IV infusion over 30 minutes, every 21 days, days 1 and 8). Unfortunately, 9 months after the diagnosis of the SCC, the patient passed away due to multiorgan failure related to the widespread chemoresistant disease.

## Discussion

At the present study, we reported two cases of ovarian SCC arising from MCT with two different stages of the disease and prognosis. It is well known that MT of MCT is a rare condition that occurs in less than 3% of MCT cases. Our both cases presented the same symptoms, including chronic abdominal pain and distention, loss of appetite, and weight loss. In case number 1, despite the large size of the tumor, the disease was at stage Ia and had a good prognosis; while, case number 2 was at stage IIIc of the disease with a poor prognosis, and progression of the disease occurred despite chemotherapy. As it has been mentioned in previous studies, large tumor size is one of the risk factors for aggressive behavior and poor prognosis; however, both of our cases with definitely large tumors had different stage of disease and prognosis. Also, the level of the of previously introduced tumor markers is unreliable, as in our case that had stage IIIc of disease, the serum levels of all markers were normal, and only the CA125 and ROMA were slightly elevated.

Unfortunately, the lack of specific clinical signs, tumor markers, or imaging features makes the preoperative and operative diagnosis of the MT very difficult, or even impossible; however, some risk factors are suggested, including age, imaging features and size of the tumor, serum level of some tumor markers, staging of disease, and residual tumors [2, 3]. In the previous studies, the mean age of the patients at the time of the diagnosis was about 55 years old, and postmenopausal women were the most group at the risk of malignancy. Our cases also were postmenopausal, 51, and 53 years old women. The most common symptoms reported in the previous case studies were abdominal pain, bloating, and a feeling of an abdominal mass [4]. In our cases also, the chief complaint was abdominal pain, loss of appetite, and weight loss. According to some studies, tumors larger than 99 mm are associated with an increased risk of MT [5], and tumors larger than 150 mm, are associated with more aggressive behavior [6]. In our cases, the tumor size was 130 × 110 × 50 mm and 140 × 90 × 65 mm.

Studies have shown that serum levels of some tumor markers, such as SCC, CA125, CEA, and CA19-9, might be higher than the normal range in this malignancy, but there is no relationship between the level of these tumor markers and tumor size or stage of the disease; however, it can be associated with worse prognosis [6]. In our study, in case number 1, despite the early stage (Ia) of the diseases and a good prognosis, serum levels of the CA125 and CEA were above the normal range, while in case number 2, who was at stage IIIc of the disease, the serum levels of the CEA, CA19-9, and HE4 were normal, and only the CA125 and ROMA were slightly elevated above the normal range. We did not measure the level of SCC antigen.

Previous studies have reported that imaging characteristics most suggestive of malignancy are the presence of solid components and involvement of adjacent organs [7]. In our cases also, the preoperative imaging showed a large cystic mass with a solid component in case number 1, but in case number 2, an irregular border and adhesion to the intestine and anterior abdominal wall were reported, in addition to the solid component.

According to the literature, the stage of the disease is the most important prognostic factor for the overall survival rate [2, 6, 8]. The 2-year and 5-year survival rate are 0–30%, and 0% respectively in cases that the disease has spread beyond the ovary (stage II–IV), while in stage I is 95%. This finding indicates that the survival rate depends on the time of diagnosis, and the onset of treatment at earlier stages.

Other risk factors that can influence the prognosis are the presence of ascites, capsule invasion, vascular invasion, rupture, and adhesion [9]. In our study, case number 2, who was in stage III c, had almost all risk factors for poor prognosis, except vascular invasion, and she had a shorter survival, but case number 1, who had none of the mentioned risk factors, showed a better outcome and prognosis.

Regarding retrospective design and limited data available in previous studies, there are still no agreements on the effectiveness of a specific treatment like adjuvant chemotherapy or radiotherapy in this disease. According to a recently published systematic review, it seems that early hysterectomy and platinum-based chemotherapy could be associated with better survival; however, this result should be interpreted with caution [10]. The most common chemotherapy regimen used in these patients is the same used for ovarian epithelial cancers, but despite multidisciplinary management, there are still a high recurrence and mortality rate [11, 12]. Patni *et al.,* similarly to our study, has used the combination of carboplatin and paclitaxel regimen [13]. Our first case took four courses of chemotherapy. In the second case, tumor growth despite three courses of chemotherapy revealed the tumor's resistance to chemotherapy and its aggressive behavior.

## Conclusion

We should remind that the diagnosis and treatment of SCC arising from MCT is still a diagnostic and therapeutic puzzle, especially in women with advanced disease, since, despite extensive initial surgery and optimal debulking, the prognosis in these patients is poor. We need to gather patient information internationally and share our experiences about this disease to reach a standard treatment protocol. Therefore, relying on our experiences and previous reports, we must remind that postmenopausal women with large dermoid cysts are more likely at risk of developing malignancy.

## Data Availability

Not applicable.

## Abbreviations

MCT: Mature cystic teratoma
MT: Malignant transformation
SCC: Squamous cell carcinoma
IUD: Intrauterine device
PET/CT: Positron emission tomography/CT scans
